# Larval nutrition differentially affects adult fitness and *Plasmodium* development in the malaria vectors *Anopheles gambiae* and *Anopheles stephensi*

**DOI:** 10.1186/1756-3305-6-345

**Published:** 2013-12-10

**Authors:** Willem Takken, Renate C Smallegange, Antoine J Vigneau, Valerie Johnston, Margaret Brown, A Jenny Mordue-Luntz, Peter F Billingsley

**Affiliations:** 1 Laboratory of Entomology, Wageningen University, PO Box 8031, 6700, EH Wageningen, The Netherlands; 2 Current address: Wageningen Academic Publishers, PO Box 220, 6700, AE Wageningen, The Netherlands; 3 School of Biological Sciences, University of Aberdeen, Tillydrone Avenue, Aberdeen AB24 2TZ, Scotland UK; 4 Current address: 18 Jasmine Court, CB1 8BG Cambridge, UK; 5 Current address: School of Pharmacy, Robert Gordon University, Schoolhill, Aberdeen AB10 1FR, UK; 6 Current address: Sanaria Inc, 9800 Medical Center Drive, Rockville, MD 20850, USA

**Keywords:** *Anopheles gambiae sensu stricto*, *Anopheles stephensi*, Mosquito, *Plasmodium yoelii nigeriensis*, Blood-feeding, Body size, Fitness

## Abstract

**Background:**

Mosquito fitness is determined largely by body size and nutritional reserves. *Plasmodium* infections in the mosquito and resultant transmission of malaria parasites might be compromised by the vector’s nutritional status. We studied the effects of nutritional stress and malaria parasite infections on transmission fitness of *Anopheles* mosquitoes.

**Methods:**

Larvae of *Anopheles gambiae sensu stricto* and *An. stephensi* were reared at constant density but with nutritionally low and high diets. Fitness of adult mosquitoes resulting from each dietary class was assessed by measuring body size and lipid, protein and glycogen content. The size of the first blood meal was estimated by protein analysis. Mosquitoes of each dietary class were fed upon a *Plasmodium yoelii nigeriensis*-infected mouse, and parasite infections were determined 5 d after the infectious blood meal by dissection of the midguts and by counting oocysts. The impact of *Plasmodium* infections on gonotrophic development was established by dissection.

**Results:**

Mosquitoes raised under low and high diets emerged as adults of different size classes comparable between *An. gambiae* and *An. stephensi*. In both species low-diet females contained less protein, lipid and glycogen upon emergence than high-diet mosquitoes. The quantity of larval diet impacted strongly upon adult blood feeding and reproductive success. The prevalence and intensity of *P. yoelii nigeriensis* infections were reduced in low-diet mosquitoes of both species, but *P. yoelii nigeriensis* impacted negatively only on low-diet, small-sized *An. gambiae* considering survival and egg maturation. There was no measurable fitness effect of *P. yoelii nigeriensis* on *An. stephensi.*

**Conclusions:**

Under the experimental conditions, small-sized *An. gambiae* expressed high mortality, possibly caused by *Plasmodium* infections, the species showing distinct physiological concessions when nutrionally challenged in contrast to well-fed, larger siblings. Conversely, *An. stephensi* was a robust, successful vector regardless of its nutrional status upon emergence. The data suggest that small-sized *An. gambiae*, therefore, would contribute little to malaria transmission, whereas this size effect would not affect *An. stephensi.*

## Background

Lifetime fitness of adult female mosquitoes is influenced strongly by nutrition during larval development, the availability and quality of blood meals, and ambient conditions. Larval nutrition determines metabolic reserves and the size of adult mosquitoes upon emergence [1-3] and superimposed on body size, the number of eggs is largely governed by the size of the preceding blood meal [4]. Anopheline larvae that develop in overcrowded conditions or suffer nutritional stress result in adult mosquitoes that may require two or more blood meals before they can initiate oogenesis and lay eggs [2]. These pre-gravid mosquitoes [5] feed soon after emergence and are likely to return for a blood meal the next day. This state is common in natural populations of *Anopheles gambiae*, with as many as 70% of the females emerging as pre-gravid adults [5]. However, the pre-gravid condition is less common in other anophelines, perhaps with the exception of *An. funestus*[1,6]*.*

Malarial parasites are often considered detrimental to survival and gonotrophic development in anophelines (reviewed by [7,8] and [9]). It is inferred that responses initiated by the female mosquito following an infectious blood meal negatively affect survival and vitellogenin deposition in the oocytes [10,11]. However, while *Plasmodium*-infected mosquitoes may exhibit reduced longevity and fecundity, the interaction of these effects with fitness upon emergence (i.e. viable reproductive output) remain unexplored [12,13].

Here we investigate the effects of larval nutrition upon adult characteristics on emergence of *An. gambiae* and *An. stephensi*. We also examine the effects of different larval nutritional regimes on mosquitoes uninfected or infected with the rodent malaria parasite, *Plasmodium yoelii nigeriensis*. We demonstrate that the two mosquito species have differing ecophysiological strategies and show that malaria transmission is possibly severely compromised in nutritionally deficient *An. gambiae*.

## Methods

### Mosquitoes

*Anopheles gambiae sensu stricto* (Suakoko strain) and *An. stephensi* (SDA500) were kept at 27°C, 80% RH, and 12 h scotophase, with 30 min dusk and dawn periods. Larvae were raised in 2.5 L of tapwater (22 x 34 cm surface area, 3.3 cm depth) and fed powdered Tetramin® fishfood (Tetrawerke, Melle, Germany) daily. Pupae were placed in 30 cm^3^ gauze cages for adult emergence. Adult males and females were kept together and provided with 8% fructose *ad libitum*. For colony maintenance, female mosquitoes were blood-fed on a human arm (*An. gambiae*) or an anaesthetised mouse (*An. stephensi*) twice per week. Eggs were collected on moist filter paper and transferred to larval trays for hatching.

For experiments, 200 newly emerged larvae were placed each in a tray and fed 0.1 mg (low diet) or 0.3 mg (high diet) powdered Tetramin® fish food per larva per day. All pupae were collected daily and placed in emergence cages. To ensure all females were mated, 6–7 d old males from the main colony were added to females (1:1 ratio) on the day of emergence.

### Mosquito size

Adult mosquitoes <24 h old were killed by freezing, placed in small glass vials and dried for 48 h at 40°C. Mosquitoes were weighed (±0.001 mg) in a Cahn electrobalance, then one wing of each mosquito glued onto a slide, and its length from the distal end of the alula to the tip, excluding the fringe scales, was measured with an ocular micrometer (±0.03 mm).

### Metabolic reservoirs and blood meal uptake

Metabolic reserves were measured within 6 h of emergence. Glycogen and lipids were determined according to [1,14,15] and protein measured with the Bio-Rad Protein Quantitation Assay kit using Pierce’s bicinchoninic acid and bovine serum albumin as a standard [16]. For quantification of blood meal size, 5 day-old female mosquitoes were fed to repletion on a healthy, anaesthetised mouse, immobilised immediately on ice, and protein determined in individual midguts after dissection [17]. Means were compared by t-test [18] unless otherwise indicated.

### Mosquito infections with *Plasmodium*

MF1 mice were infected with *Plasmodium yoelii nigeriensis*[19] by i.p. injection of 5 μL infected blood. Mice were checked microscopically on day 4 p.i. for the presence of parasites and exflagellating gametocytes. If exflagellations were observed, experimental mosquitoes were fed upon the anaesthetised mice.

Paired groups of approximately 30 3-day old females of low- and high-diet mosquitoes of the same species were fed simultaneously to repletion on one infected mouse. Fed mosquitoes were maintained as above for five days, cold-immobilised and midguts examined for oocysts after mercurochrome staining [20]. Prevalence is defined as the proportion of mosquitoes infected with *Plasmodium*; intensity of infection is defined as the geometric mean number of oocysts per mosquito.

### Ovarian development

Mosquitoes of both diet regimens that had fully engorged on uninfected and *Plasmodium*-infected mice were dissected in physiological saline 3 days after the blood meal. Ovaries were examined at 400x magnification under a dissecting microscope. Development of ovaries was scored according to [21], and the number of developing eggs in each ovary, if applicable, was counted.

### Statistical analysis

The effects of larval diet on adult body size and metabolic reserves were analysed with a Generalized Linear Model (GLM; Genstat, release 6.1). Two-sided t-probabilities were calculated to test pairwise differences between means. Effects were considered to be significant at p < 0.05. GLMs were also used to examine the effect of body size on bloodmeal uptake, and the effect of body size and *Plasmodium* infections on gonotrophic development. All possible two-way interactions were examined. The effect of body size on mosquito survival and on the proportion of oocyst-infected mosquitoes were analysed by GLM using a binomial distribution [20]. The number of oocysts per mosquito within a group were log-transformed and analysed as Poisson-distributed data, and GLM used to investigate the effect of body size on oocyst infections [18,22].

### Ethics statement

All animal work described in this paper was approved by the University of Aberdeen ethical review committee and was performed under an approved United Kingdom Home Office project licence (Licence Number PPL 60 2810).

No human subjects were asked to participate in this study and ethical clearance was not required. Although mosquitoes were fed upon a human arm, this was performed only by the first author as a standard routine method for mosquito maintenance.

## Results

### Wing length, dry weight and metabolic reserves

**Figure 1:**
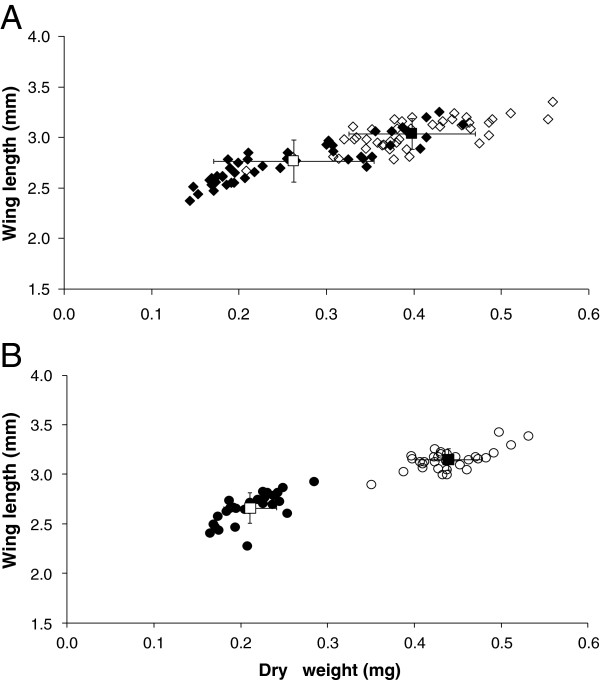
**Relationship between dry weight (mg protein) and wing length (mm) in *Anopheles gambiae *(A) and *Anopheles stephensi *(B).** Each point represents a single mosquito. Open symbols, high diet; solid symbols, low diet. Error bars represent SEM.

As the study focused on the blood meal uptake and *Plasmodium* infection, only female mosquitoes were analysed. The low and high larval diets produced significantly different size classes of mosquitoes (p < 0.001). Mean wing lengths for low and high diet *An. gambiae* were 2.77 ± 0.03 mm (n = 55) and 3.03 ± 0.02 mm (n = 48), and for *An. stephensi* were 2.66 ± 0.03 mm (n = 32) and 3.15 ± 0.02 mm (n = 35), respectively. The low diet *An. gambiae* (range 2.37-3.25 mm) included a number of individuals with dry weights and wing lengths comparable to those seen in the high-diet group (range 2.67-3.35 mm) and there was a poorer demarcation between low- and high-diet groups in this species (Figure 1).

**Figure 2:**
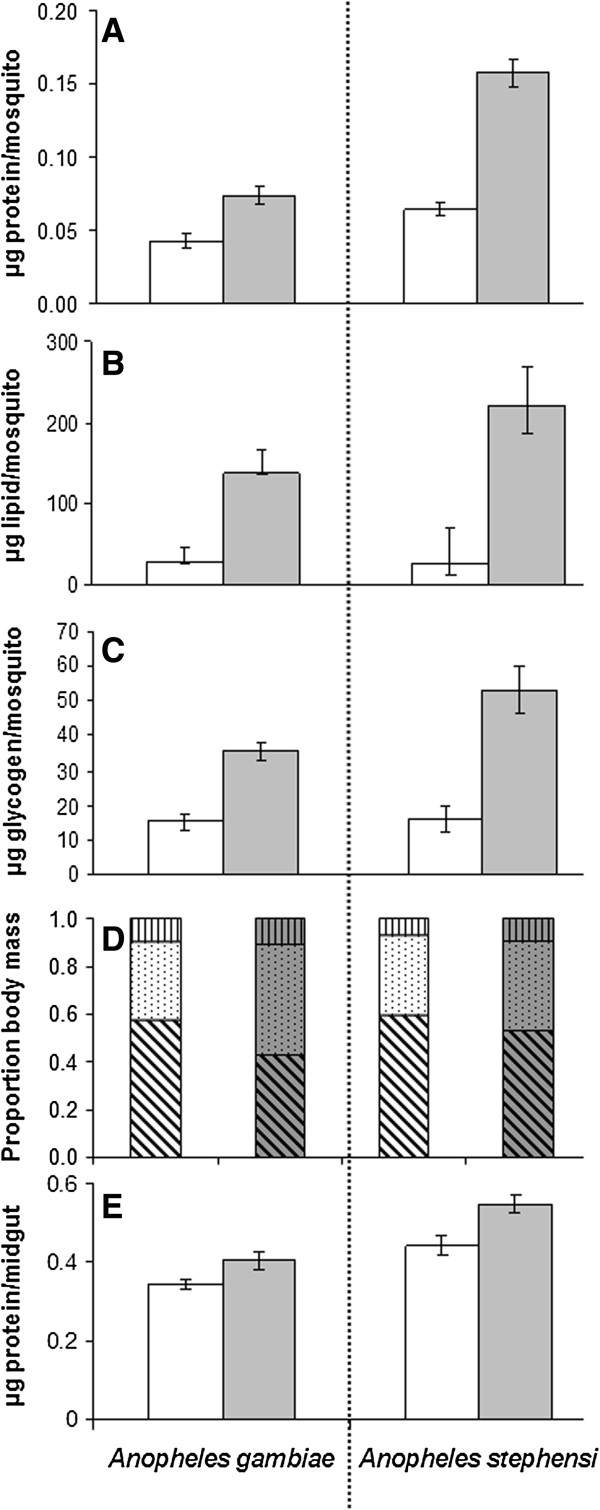
**Effect in *Anopheles gambiae *(left) and *Anopheles stephensi *(right) of larval diet regime on total body reserves of protein (A) (n = 10 per treatment), lipid (B) (n = 30) and glycogen (C) (n = 10), on their relative proportion (D) upon emergence (means from A-C calculated as a proportion of dry weight, n = 28-48), and on blood meal size (E) (n = 10 except in low-diet *An. gambiae*, where n = 5).** White background, low diet; grey background, high diet. In **(D)**, vertical shading is glycogen, stippled shading is lipid and diagonal shading is protein. Error bars represent SEM.

Low-diet mosquitoes of both species had significantly less protein, lipid and glycogen upon emergence than high-diet individuals (p < 0.001) (Figure 2A-C). Corrected for dry body weight, the high-diet mosquitoes had proportionally significantly (p < 0.05 for all comparisons) more protein, lipid and glycogen reserves than the low-diet ones (Figure 2D). Blood ingestion per female increased significantly in high-diet mosquitoes (p < 0.001) from 0.344 to 0.405 mg protein /midgut in *An. gambiae* and from 0.359 to 0.548 mg protein/midgut in *An. stephensi*. While the increase in blood uptake between high- and low-diets groups was greater in *An. stephensi* than in *An. gambiae*, the relative increase was approximately 1.2-fold in both species (Figure 2E).

### *Plasmodium* infections and survival

**Figure 3:**
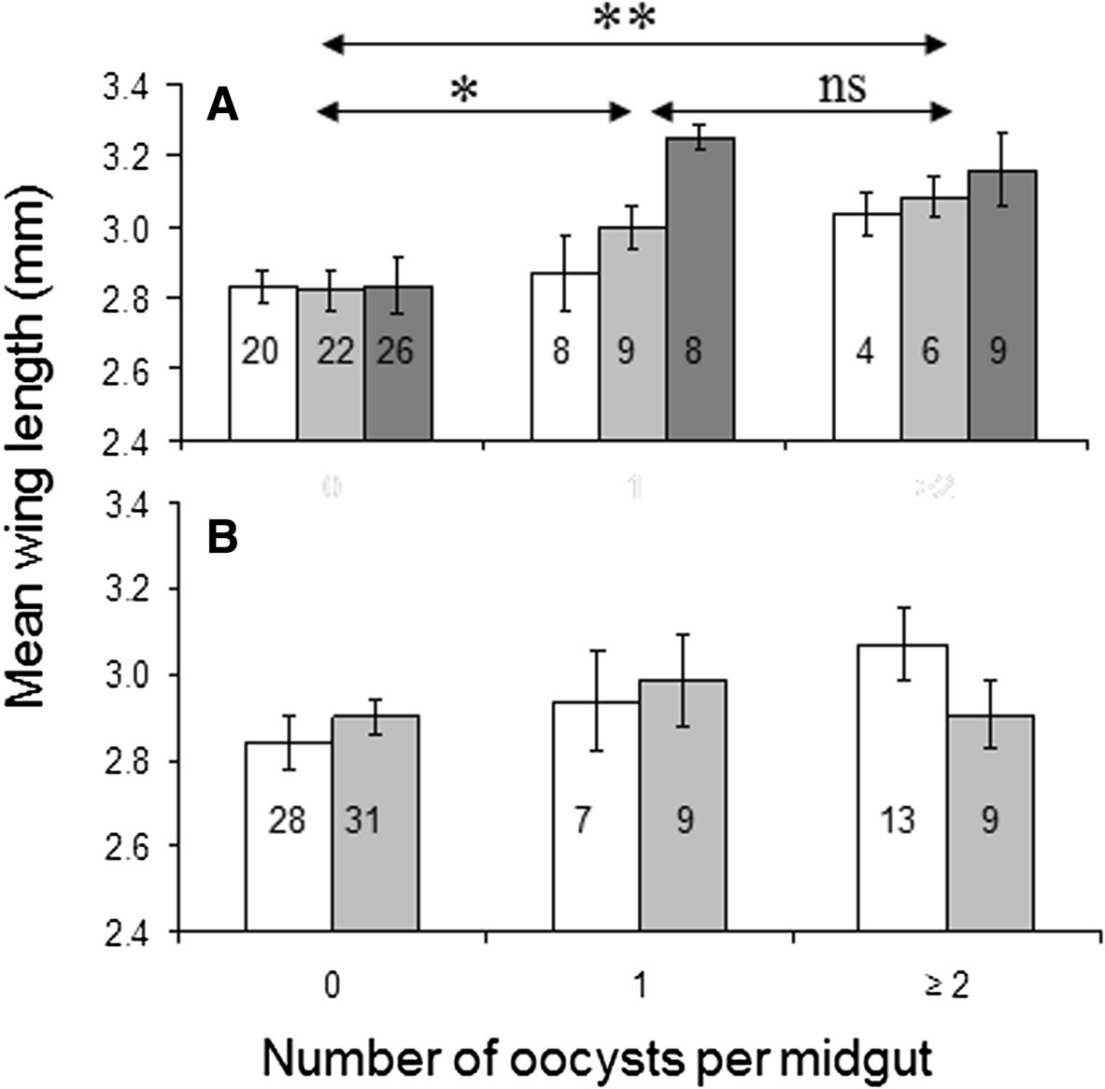
**Size of *Anopheles gambiae *(A) and *Anopheles stephensi *(B) mosquitoes infected at different intensities by oocysts of *Plasmodium yoelii nigeriensis*.** Differently coloured bars represent replicates ± SEM. Sample sizes are shown within the bars.

We were able to obtain infection rates of *P. y. nigeriensis* much more comparable to those seen in the field for *P. falciparum* than usually reported in studies of murine malaria transmission [20,23]. Only 24% of the low-diet *An. gambiae* given an infectious blood meal survived the 5 days until oocysts developed (Table 1). The mean oocyst prevalence of this group was 0.10 and the mean intensity ranged from 0 to 0.18 oocysts per mosquito. Of the high-diet *An. gambiae*, 75% survived 5 days after feeding. The mean oocyst prevalence was significantly higher (p < 0.001) at 0.49 and the mean intensities ranged from 0.59-0.87 oocysts per mosquito (p < 0.005 compared to low diet). Survival rates of low-diet *An. gambiae* were significantly lower than of the high-diet group (p < 0.001). There was no significant correlation of oocyst infections with body size but uninfected females were significantly smaller than mosquitoes infected with one (p = 0.01) or more (p = 0.001) oocysts (Figure 3A).

**Table 1:** **Effect of body size on *Plasmodium yoelii nigeriensis *** **development in *Anopheles gambiae *** **and *An. stephensi *** **raised on different larval nutritional quantities**

		**Low diet**					**High diet**				
	**Percent parasitaemia**	***N***	**Proportion surviving**	**Number dissected**	**Oocyst infections**		***N***	**Proportion surviving**	**Number dissected**	**Oocyst infections**	
					**Prevalence**	**Intensity**				**Prevalence**	**Intensity**
*Anopheles gambiae*	8.23	45	0.18	8	0	0	67	0.76	25	0.48	0.62 (0.48-0.75)
	6.38	63	0.24	15	0.13	0.10 (0.03-0.16)	65	0.74	25	0.60	0.87 (0.72-1.02)
	6.05	61	0.30	17	0.18	0.13 (0.06-0.20)	55	0.76	25	0.40	0.59 (0.46-0.74)
*Mean*			0.24		0.10			0.75		0.49	
*Anopheles stephensi*	20.19	27	0.93	25	0.24	0.22 (0.14-0.30)	33	0.82	24	0.58	1.57 (1.34-1.80)
	8.27	26	0.96	22	0.41	0.57 (0.42-0.72)	37	0.97	28	0.36	0.60 (0.43-0.78)
*Mean*			0.94		0.32			0.90		0.47	

*N* – number of mosquitoes that took an infectious blood meal.

Mean survival rates of *An. stephensi* were ≥90% regardless of larval diet. Mean oocyst prevalence increased from 0.32 in the low-diet mosquitoes to 0.47 in the high-diet group and the intensity of infections similarly increased (Table 1). The oocyst intensities in *An. stephensi* were higher than in *An. gambiae* in both size classes. There was no difference in mean wing length of uninfected or infected *An. stephensi* (Figure 3B).

### *Plasmodium* infections and gonotrophic development

**Figure 4:**
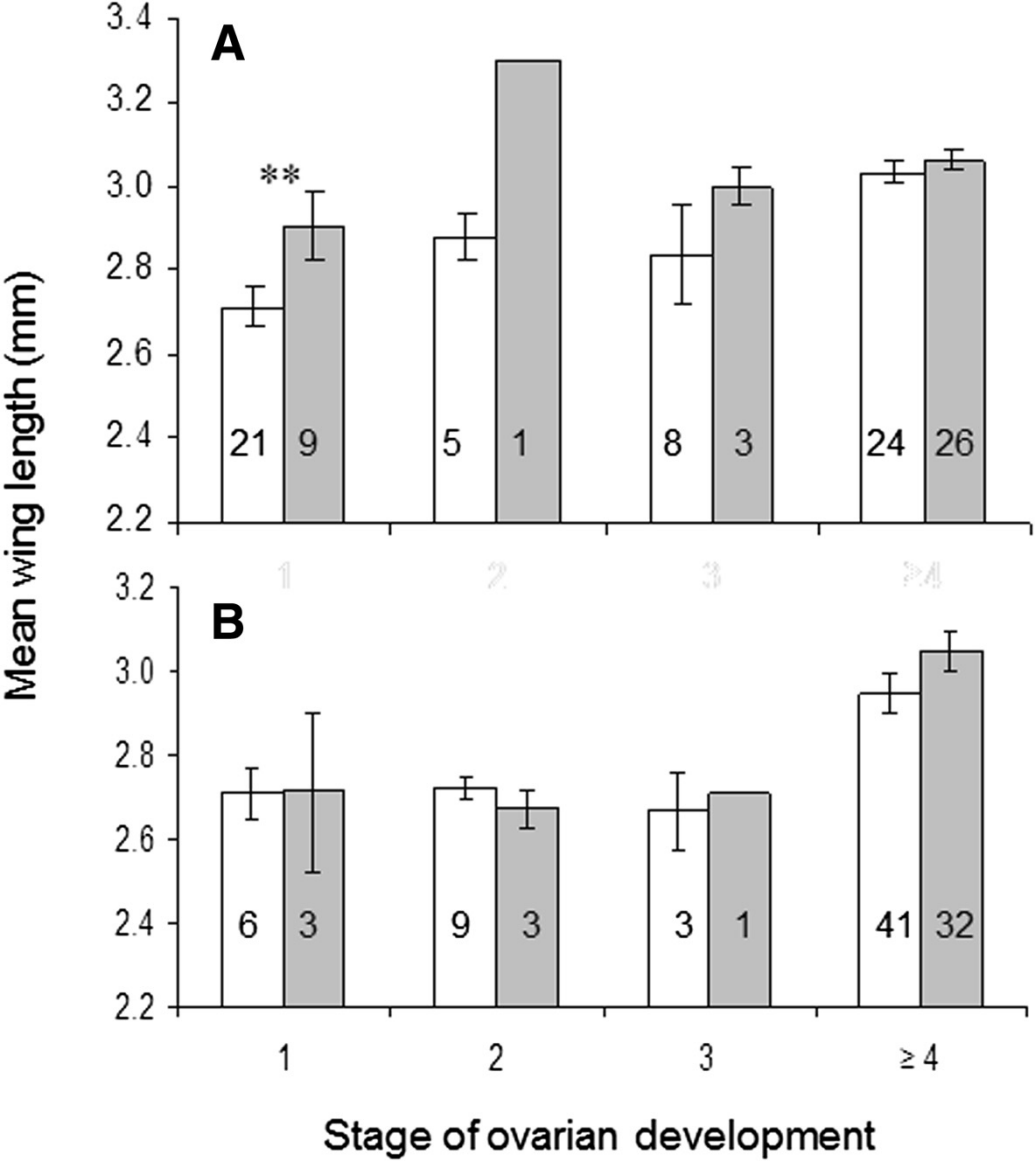
**Size of uninfected (white) and *Plasmodium yoelii-*infected (grey) *Anopheles gambiae *(A) and *Anopheles stephensi *(B) reaching different stages of ovarian development five days after feeding.** Error bars represent SEM. ** - significant difference between uninfected and infected, p < 0.01. Mean size of uninfected *An. gambiae* at stage ≥ 4 was significantly greater than those at stages 1 (p < 0.001) and 3 (p = 0.008). Mean sizes of both groups of *An. stephensi* at stage ≥ 4 were significantly (p < 0.05) greater than stages 1 and 2.

**Figure 5:**
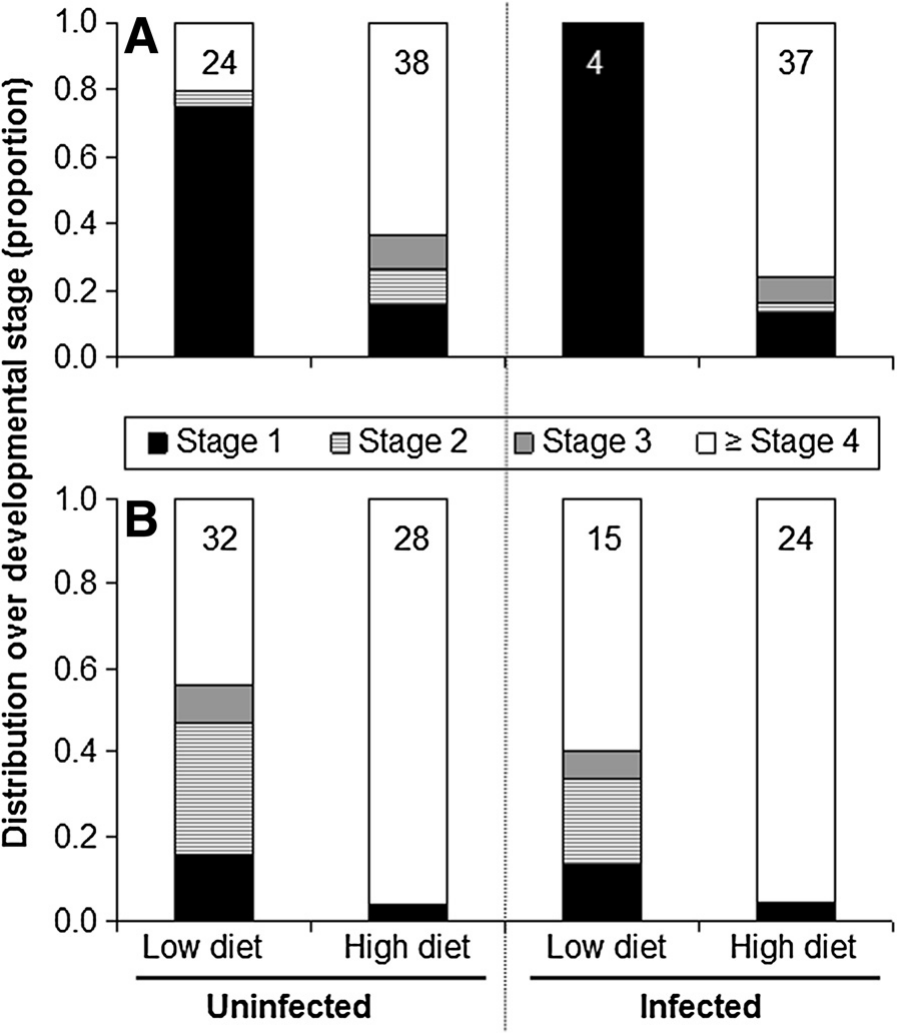
**Effect of a single blood meal to repletion and *Plasmodium yoelii nigeriensis *** **infection on gonotrophic development in *Anopheles gambiae *** **(A) and *Anopheles stephensi *** **(B) fed low and high diets.**

Parasite infections had little effect on ovarian development in both mosquito species. High-diet, larger mosquitoes, both uninfected or infected, matured eggs more successfully than individuals raised on the low diet (p < 0.01), except in infected *An. gambiae* (Figure 4). Parasite infections had a greater effect on gonotrophic development in *An. gambiae* than in *An. stephensi*, as the proportion of infected female *An. gambiae* with reduced gonotrophic development (Christophers stages I-II) was larger than that of uninfected females (stage I: p = 008; stage II: p = 0.036). *Plasmodium*-infected, low-diet *An. gambiae* did not initiate oogenesis, while uninfected and infected rich-diet females were at similar stages of ovarian development 5 days after the infectious blood meal. In *An. stephensi* there was no effect of *P. yoelii* infection on ovarian development in both size classes (Figure 5).

## Discussion

The nutritional status of larvae of *An. gambiae* and *An. stephensi* is manifested as differences in body size and reserves of protein, lipid and glycogen in teneral adults. Nutritionally-stressed *An. gambiae* emerge as small individuals that die within 48 h following emergence if deprived of a carbohydrate source [2,24]. Well-fed individuals survive longer. Upon emergence, the fitness of mosquitoes is related directly to size, and in our study the quantity of larval food and not larval density caused these effects. Nutritionally-deprived larvae required 2–3 more days to reach pupation than the nutritionally well-fed siblings (data not shown). Both species responded in a similar fashion to the different larval diets, although in *An. gambiae* there was a gradient of adult size so that low-diet and high-diet size classes were not as distinct as those in *An. stephensi*. This may be because the more competitive larvae of *An. gambiae* were able to ingest more food to the detriment of their siblings or due to cannibalism [25]. Alternatively, at the larval density chosen for this study, density-dependent effects may already have been present in *An. gambiae* and not in *An. stephensi*[26]. Okech *et al.*[13] demonstrated the importance of nutrients from natural breeding sites on body size of *An. gambiae*. Although the nutrients used in their study were different from those in the present study, the data corroborate our findings about nutritionally-deprived mosquitoes.

Mosquito body size affects reproduction and survival, with larger mosquitoes producing more eggs during their lifetime than their smaller counterparts [1,27,28]. This is the case for both *An. gambiae* and *An. stephensi* as the gonotrophic development of the low-diet class of mosquitoes was much reduced compared with that of the high-diet class. The proportion of low-diet females entering a pre-gravid stage was greater than in the high-diet group, in which such a physiological condition was low or absent. Pre-gravid behaviour of wild *An. gambiae* is well documented [5,12,29], the proportion of adults in this condition varying seasonally, possibly depending on conditions in larval habitats. As we demonstrate in the present work, this may profoundly affect the ability of the mosquitoes to transmit malaria parasites. No such pre-gravid physiology was observed in *An. stephensi*.

Although we expected that the low diet would preferentially compromise lipid storage [1,30], the deposition of protein, lipid and glycogen were all affected. Hence, low-diet mosquitoes not only started adult life as small individuals, but were also severely compromised nutritionally; this is manifested in a higher mortality rate in both laboratory [2,24] and field [12] studies. The need for blood meals to aid adult nutrition rather than just reproduction is thus important. Additional protein required by small adults for survival must be derived from vertebrate blood, as the insects have limited protein reserves upon emergence [2,31]. Conversely, nutritionally-rich larval diets are multiply beneficial; with increased body size and reserves, female mosquitoes not only live longer but ingest larger blood meals, complete ovarian development more successfully (this paper) and compete better for males [32,33].

The biological interaction between nutritional status and *Plasmodium* infections is intriguing. In a large mosquito, uptake of a larger infectious blood meal will result in more parasites entering the mosquito midgut [34]. This could be harmful as higher death rates of large mosquitoes has been attributed to increased oocyst load that in turn competed for nutritional resources [35] or induction of a damaging immune response [36]. Our study is in agreement with the findings of Okech *et al.*[13] that body size affects the number of parasites that develop into oocysts. As a general rule, small mosquitoes developed fewer oocysts than their larger siblings. Although it is tempting to relate this observation to the smaller quantity of blood ingested by smaller females, this is clearly not the sole explanation. In *An. stephensi*, a 1.3-fold increase in blood meal size from low- to high-diet groups was accompanied by a similar 1.5-fold increase in prevalence and a modest 2.8-fold increase in mean oocyst intensity. Thus, in this species, differences in blood meal size probably account for all the differences in oocyst rates from the same infectious blood source. In stark contrast, the increase in blood meal size from low- to high-diet *An. gambiae* resulted in an approximately 5-fold increase in prevalence and 9-fold increase in mean intensity. Clearly the differences in blood meal size cannot alone account for the differences in oocyst infections.

Many more gametocytes are ingested during a blood meal than can be accounted for as oocysts [37,38]. The likely candidates for modulating infections are components of the mosquito immune response [39-41]. Indeed, oxidative stress of the mosquito contributes greatly to the ‘infectious physiological background’ into which the gametocytes are placed during feeding [42,43]. The dramatic differences in infection rates between low- and high-diet mosquitoes suggest that defensive mechanisms against ookinetes may be stimulated by the low diet, and when parasites are ingested by these mosquitoes, they either overload the already burdened stress responses to kill the mosquito or are killed by it. In contrast, large mosquitoes are less stressed upon emergence and consequently can maintain low-level infections without necessarily triggering the immune response or compromising their own survival. Such a model would favour the maintenance of highly responsive and biologically expensive immune genes in the population at low frequencies because while most of the small, stressed mosquitoes will die young, those that do survive may still contribute one or two batches of eggs to the population.

Our results suggest that parasites effected a greater mortality on the mosquitoes in nutritionally-compromised *An. gambiae* than in well-fed ones. In this group, although we did not study the mortality of uninfected mosquitoes, Lambrechts *et al*. [44] report a significant parasite-effected mortality in *An. stephensi* by *P. yoelii*. Similarly, Dawes *et al*. [9] report a density-dependent effect of *P. berghei* ookinetes in *An. stephensi*. Other reasons could be the small blood meal size or the absence of a critical factor for the oocyst. In a recent field study, investigating the effect of natural *Plasmodium* infections on wild *An. gambiae* and *An. arabiensis*, mosquitoes with larger body size produced more eggs but sporozoite infection did not affect egg production, which corroborates our findings [45]. As with our study, small-sized individuals may not have been noticed as they died early in the infection process possibly due to reduced fitness [35]. From this we conclude that mosquito fitness contributed to the higher survival rate of well-fed *An. gambiae. Plasmodium yoelii nigeriensis* had only a small effect on *An. stephensi* of both size classes, which may be attributed to the relatively low oocyst load of infected mosquitoes.

Parasite infections had only a negligible effect on the gonotrophic development in both species, in contrast to previous demonstrations of *P. y. nigeriensis* negatively affecting fecundity of *An. stephensi*[19,44,46]. However, occyst intensities in the present study were <1, whereas much greater oocyst burdens were used previously. It is therefore possible that oocyst load directly affects mosquito fitness and that the densities observed in our study were below the threshold for negative effects to occur. The above-mentioned studies added para-aminobenzoic acid (PABA) to sugar water as an extra nutritional source following a *Plasmodium*-infected blood meal. PABA may have affected the infectiousness of the parasites, leading to relatively high oocyst intensities, which are unusual in wild mosquitoes [20,23]. Natural densities of *P. falciparum* oocysts typically average 0.25-3 oocysts per mosquito (range = 0–60) [20,47-49] and this strong skew in favour of no infections and low densities may serve as a natural mechanism or trade-off, regulating mosquito fitness in response to *Plasmodium*. With low oocyst densities there was no effect of *P. y. nigeriensis* on ovarian development in *An. stephensi*; body size was the only factor determining fecundity. Hopwood *et al.*[50] reported significant apoptosis and ovarian resorption in *An. stephensi* as a result of *Plasmodium* infection. We did not observe negative effects of parasite infections on ovarian development, possibly because the oocyst intensities were lower or because we had not added antibiotics and para-aminobenzoic acid to the adult sugar source. In *An. gambiae* the parasites affected the small-sized mosquitoes: mortality of infected mosquitoes increased and ovarian development was stopped. However, because so few low-diet mosquitoes survived the oocyst incubation time (n = 4), the results are equivocal and further examination is required. Gonotrophic development of large females of *An. gambiae* was unaffected by parasite infections. It is possible that in previous studies the combination of large-sized mosquitoes and high parasite densities may have obscured any influence of diet, environment or parasite on mosquito fitness [51].

These studies emphasise the important differences in the physiological adaptations to nutritional stress of two closely-related mosquito species. Under identical conditions, both species respond to dietary quantity and availability by altering their body size and nutrional reserves. However, while *An. gambiae* fitness is severely compromised by the low-diet, small and large *An. stephensi* are of comparable fitness in the short period of study. The reasons for this are unknown, but may result from the disproportionately poor lipid storage in small *An. gambiae*, conferring poor fitness.

The low infection rates and low impact of the parasite on high-diet mosquitoes may more closely reflect natural interactions between malaria and mosquitoes. Mosquito fitness is regulated in the larval stages by density dependence and environment [12], and in the adult stages by climate and the availability of blood sources, carbohydrate resources and oviposition sites [2,52,53]. Parasite infections may have important fitness consequences for the vector [7,54], and the scale of these consequences will be controlled by the vector and parasite interaction. The evolutionary co-adaptation of parasite and vector will eventually determine the success of transmission, and from our results it appears that a propensity for low parasite infections may be the best adaptive strategy for this interaction.

## Conclusions

The nutritional status of *An. gambiae* and *An. stephensi* upon emergence had different outcomes upon *Plasmodium* infections. Nutritionally-compromised females of *An. gambiae* died early in adult life, had a lower prevalence of *Plasmodium* infection and developed significantly fewer oocysts than well-fed females. In contrast, the nutritional status of *An. stephensi* had no effect on survival over the period of study nor on *Plasmodium* development. In both mosquito species poor nutrition but not *Plasmodium* infection inhibited ovarian development.

## Competing interests

The authors declare that they have no competing interests.

## Authors’ contributions

Conceived and designed the experiments: WT PFB JM-L. Performed the experiments: WT AJV VJ MB. Analyzed data: WT RCS PFB. Wrote the paper: WT and PFB. All authors read and approved the final version of the manuscript.
